# Chemical Composition and Mechanism of Vibriocidal Action of Essential Oil from Resin of *Protium heptaphyllum*

**DOI:** 10.1155/2019/9563213

**Published:** 2019-10-23

**Authors:** Josiane Lima Mendes, Thiago Ferreira de Araújo, Mário Geraldo de Carvalho, Francisco Eduardo Aragão Catunda Júnior, Renata Albuquerque Costa

**Affiliations:** ^1^UNINTA College (UNINTA), Sobral, Ceará, Brazil; ^2^State University of the Tocantina Region of Maranhão, Imperatriz, Maranhão, Brazil; ^3^Chemistry Department, Federal Rural University of Rio de Janeiro, Seropédica, Rio de Janeiro, Brazil

## Abstract

*Protium heptaphyllum* is a plant widely distributed in Brazilian ecosystems that produce a resin which has pharmacological activities. In this study, the chemical composition, antimicrobial and antibiofilm activity, and the possible mechanism of action against the bacterium *V. parahaemolyticus* of essential oil from *P. heptaphyllum* (EOPH) were investigated. Twenty-two components were detected in EOPH, and *β*-phellandrene (60.68%) had the majority. The inhibition halo, MIC, and MBC values were 11 mm, 2 mg/mL, and 8 mg/mL, respectively. Biofilm biomass inhibition and biomass reduction of the preformed biofilm were detected at 4 mg/mL EOPH concentration. The assays of cell constituent release and membrane permeability indicated that EOPH may disrupt the cell membrane, leading to leakage of intracellular constituent as reducing sugars and materials with an absorbance of 260 nm.

## 1. Introduction

*Protium heptaphyllum* (Aubl.) is a tree which belongs to the Burseraceae family that produces an amorphous and aromatic resin with pharmacological activities in nervous and immunological systems and gastrointestinal tract [1]. In addition, it is used in folk medicine as an analgesic and anti-inflammatory agent, in healing, and as an expectorant, which is rich in pentacyclic triterpenes and essential oils [2].

This species of phanerogama is widely distributed in Brazilian ecosystems, such as Cerrado and Amazon rainforest. With the chemical composition rich in terpenes, the essential oil obtained from *P. heptaphyllum* resin (EOPH) has been studied due to its potential antibacterial effect [3]; however, the effect against the *Vibrio parahaemolyticus* species is still incipient.

*V. parahaemolyticus* is a human enteropathogenic bacterium and is also pathogenic to shrimp and finfish [4]. This species produces the thermostable direct hemolysin (TDH), which is the sole cause of the Kanagawa phenomenon (KP), a special *β*-type haemolysis in the Wagatsuma agar. TDH also exerts several other biological activities; the major includes lethal toxicity, cytotoxicity, and enterotoxicity [5].

*V. parahaemolyticus* cause vibriosis—infections normally acquired through exposure to sea water or through consumption of raw or undercooked contaminated seafood. Noncholera bacteria can lead to several clinical manifestations, most commonly mild, self-limiting gastroenteritis. The incidence of vibriosis is rising, perhaps owing in part to the spread of *Vibrio* spp. favoured by climate change and rising sea water temperature [6].

In this study, the EOPH was analyzed to investigate chemical composition and antimicrobial and anti-biofilm activity against *V. parahaemolyticus*.

## 2. Materials and Methods

### 2.1. Essential Oil

The essential oil from the resin of *P. heptaphylum* (EOPH) was obtained by steam extraction from Brazilian plants and purchased in April 2018 from the company Laszlo (Minas Gerais, Brazil). EOPH was used directly (concentrated) or dissolved in tryptone soy broth (Difco) culture media with 1% of Tween 80. The vial containing 10 mL of the EOPH was kept refrigerated (8°C) until analysis began in August 2018.

### 2.2. Bacterium and Culture Media

The strain of *V. parahaemolyticus* serotype K 15 isolated from an outbreak of gastroenteritis that occurred in Cascavel (CE) in 1975 [7] was provided by the Laboratory of Environmental Microbiology and Fish of the Institute of Marine Sciences of the Federal University of Ceará. The strain was stocked in skim milk (Difco) with glycerol and was reactivated in tryptone soy broth (TSB) (Difco) containing 1% NaCl, incubated at 37°C for 24 hours.

### 2.3. Analysis by Gas Chromatography Coupled to Mass Spectrometry (GC-MS)

A qualitative analysis of the chemical composition of the EOPH by gas chromatography coupled to mass spectrometry (GC-MS) was carried out on a Shimadzu model spectrophotometer, model QP-2010 (Kyoto, Japan), operating with an ionization energy of 70 eV. A DB-5MS polymethylsiloxane fused silica capillary column (30 m in length, an internal diameter of 0.25 mm, and 1 *μ*m thick film) (J&W Scientific In., Folsom, USA) was used and a helium gas charger with a flux of 1. The temperatures of the injector and detector were programmed at 250°C and 200°C, respectively, and transfer line temperature was 260°C. The temperature of the chromatographic oven was 70°C with a heating ramp of 4°C 1 mL/min to 180°C for 27.5 min, then by a heating ramp of 25°C mL/min to 250°C at the end of the run. Each chromatogram peak was identified by its spectrum (NIST—147,198 compounds), using sources from the literature [8] and injections of authentic standards. Kovats was performed by coinjection with standards of alkanes (C_8_ to C_30_).

### 2.4. Analysis by Gas Chromatography by Using a Flame Ionization Detector

The quantitative analysis of the chemical composition of the oil was performed by CG-DIC in a Varian CP-3380 instrument (Palo Alto, USA), with a flame ionization detector (DIC), stable phase column CP-Sil 8 CB polymethylsiloxane (30 m × 0.25 mm × 0.25 *μ*m; Varian Inc., Palo Alto, USA), 1 : 50 flow division injection mode throughout the run (30.3 min), hydrogen carrier gas with a constant flow of 1.5 mL·min^−1^, injector temperature 230°C, and detector temperature 260°C. Chromatographic furnace programming was performed as follows: initial temperature of 70°C with a heating ramp of 4°C·min^−1^ to 180°C for 27.5 min, followed by a heating ramp of 25°C·min^−1^ to 250°C, at the end of the race. The percentage of constituents was calculated by the integral of the area of the respective peaks in relation to the chromatogram registered by DIC, total area of all constituents of the sample. The constituents of the EOPH were identified by visual comparison with the NIST 08 library and by comparing retention indices with those in the literature [8].

### 2.5. Antibiogram, MIC, and MBC Assays

The antibiogram was performed by the disk-diffusion test in Müller–Hinton agar (Difco) with 1% NaCl following the recommendations of CLSI [9]. The strain after reactivation was diluted in sterile 1% saline solution and adjusted to 1.5 × 10^8^ colony-forming units per milliliter. After the inoculation, filter paper disks (6 mm diameter) were used in triplicate, soaked with 20 *μ*L of EOPH with incubation at 35°C/24 h. As a positive control, meropenem (10 *μ*g) was used. The antibacterial activity was considered when inhibition halos were ≥10 mm. For MIC and MBC [9], determination microdilution in tryptone soy broth (TSB) (Difco) using polystyrene plates of 96 wells was used. Concentration of strain was adjusted in TSB to 1.25 × 10^7^ colony-forming units (CFU·mL^−1^). Three replicates of each strain were tested with EOPH in concentrations of 16 mg·mL^−1^, 8 mg·mL^−1^, 4 mg·mL^−1^, 2 mg·mL^−1^, 1 mg·mL^−1^, 0.5 mg·mL^−1^, and 0.25 mg·mL^−1^. To determine the MBC, a pool was made of all wells for each concentration, and then plating was performed in 10 *μ*L in triplicate tryptone soy agar. MIC was determined as the lowest concentration of EOPH that was able to inhibit the visible growth of V. parahemolyticus in the plates. MBC was the lowest concentration of OEPH that killed V. parahaemolyticus and was indicated by the absence of microbial growth in tryptone soy agar [10].

### 2.6. Antibiofilm Activity

Microtiter-plate technique [11] with the crystal violet (CV) assay was used [10]. For determination of EOPH action in the biofilm, the plates were subjected to reading the optical density using a microplate reader (Molecular Devices-Spectra Max Paradigm Multi-Mode) at a wavelength of 595 nm. The activity was found in concentrations that did not observe the adhesion of crystal violet in any of the wells.

### 2.7. Controls

The controls are followed according to Vasconcelos et al. [10]. For the CIM, CBM, and antibiofilm activity, we used as turbidity control the culture medium (TSB) containing the test substance (EOPH) in concentrations of 16 mg·mL^−1^, 8 mg·mL^−1^, 4 mg·mL^−1^, 2 mg·mL^−1^, 1 mg·mL^−1^, 0.5 mg·mL^−1^, and 0.25 mg·mL^−1^. In the contamination control, we used only the TSB in three wells on the plates. The negative control was done from the inoculation of 100 *μ*L of the strain (1.25 × 10^7^ CFU·mL^−1^) in wells containing 100 *μ*L TSB.

### 2.8. Cell Constituent Release

The release of cell constituents into supernatant was analyzed according to the method described by Diao et al. [12] with some modifications. The *V. parahaemolyticus* strain was grown in 50 mL TSB (Difco) at 37°C for 24 h. After this time, the cells were collected by centrifugation (10,000 rpm for 15 minutes) (MARK) and washed 3x with 1% saline. The bacterial culture was then resuspended in the TSB medium and applied in three tests: control (medium strain only), 2x MIC, and 3x MIC, incubating at 37°C for six hours. After the incubation period, centrifugation (10,000 rpm for 15 minutes) was done, and the supernatant was used to determine reducing sugars and optical density reading at 260 nm.

### 2.9. Cell Membrane Permeability

The permeability of the cell membrane was expressed as the relative electrical conductivity according to the method described by Diao et al. [12]. *V. parahaemolyticus* in TSB (Difco) was incubated at 37°C for 10 h and centrifuged for 15 minutes to form pellets. Then, the cells were washed with 5% glucose until their electrical conductivities were near to that of 5% glucose. The EOPH in concentrations of 1x MIC, 2x MIC, and negative control was added into the isotonic bacterial solution with incubation at 37°C for 6 h. The conductivity of the culture medium was measured at following selected intervals: 0, 30, 60, 120, 240, and 360 minutes. The permeability of the bacterial membrane was calculated according to the following formula:

(1) $$\begin{array}{c} \%\text{ relative electrical conductivity}=\frac{100\times \left(L_{2}-L_{1}\right)}{L_{0}}, \end{array}$$

where *L*_0_ is the conductivity of bacteria in 5% glucose treated in boiling water for 5 minutes, *L*_1_ is the electrical conductivity of the essential oil (1x MIC and 2x MIC) and negative control in 5% glucose, and *L*_2_ is the conductivity measured at selected time intervals of isotonic bacterial solution + essential oil in concentrations of 1x MIC, 2x MIC, and negative control.

### 2.10. Statistical Analysis

Data analysis in GraphPad Prism 5 with ANOVA statistical followed by Student–Newman–Keuls test for significant variability comparisons (*p* < 0.01) between different tested concentrations was used [10].

## 3. Results

### 3.1. Chemical Composition of the Essential Oil

EOPH presented 22 constituents, and *β*-phellandrene (60.68%) was the majority (Table 1) followed by *p*-cymene (13.63), *α*-pinene (4.47), and *α*-phellandrene (3.38%).

### 3.2. Antibacterial Activity

**Figure 1:**
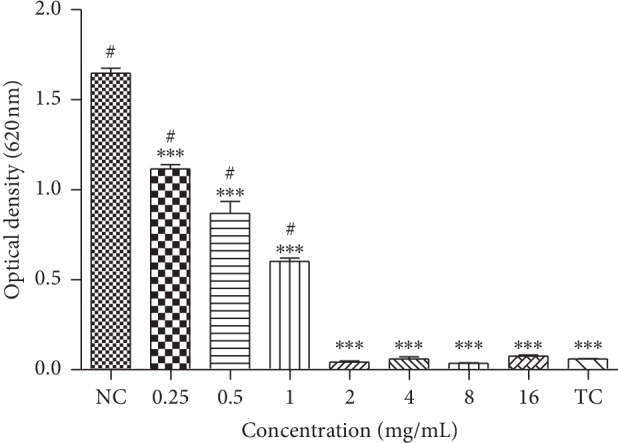
Minimum inhibitory concentration (MIC) of *Protium heptaphylum* essential oil (EOPH) against *Vibrio parahaemolyticus* determined from reading the absorbance of the bacterial culture treated with EOPH at different concentrations. NC: negative control. TC: turbidity control. ^*∗∗∗*^*p* < 0.01 vs. NC. ^#^*p* < 0.01 vs. TC.

The antibacterial activity of EOPH was confirmed by the detection of inhibition zone halos of 11 mm. This result was lower than the positive control meropenem (30 mm). MIC of 2 mg/mL (Figure 1) and 8 mg/mL of MBC were observed. EOPH shows bactericidal effect since the MBC/MIC ratio was ≤4 [13].

Figure 1 shows that concentrations of 1, 0.5, and 0.25 mg/mL were able to reduce the bacterial population of *V. parahaemolyticus* since there was a significant difference (*p* < 0.01) when compared to the negative control.

### 3.3. Antibiofilm Activity

**Figure 2:**
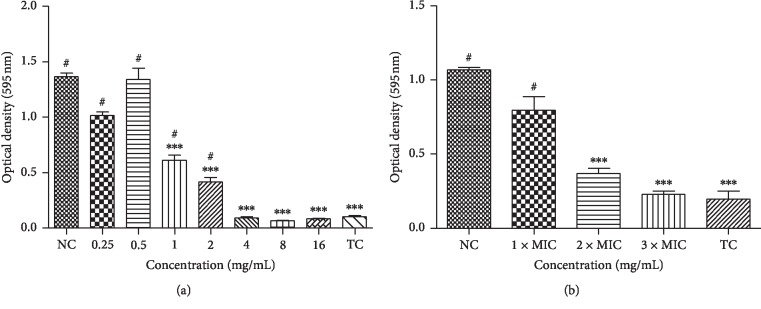
(a) Absorbance of biofilm biomass after 48 hours in contact with *Protium heptaphyllum* essential oil (EOPH) at different concentrations. (b) Absorbance of preformed biofilm after six hours in contact with EOPH at different concentrations. NC: negative control. TC: turbidity control. MIC: minimum inhibitory concentration. ^*∗∗∗*^*p* < 0.01 vs. NC. ^#^*p* < 0.01 vs. TC.

EOPH presented the ability to prevent biofilm formation of *V. parahaemolyticus* (48 h) in 4 mg/mL concentration. In addition, concentrations of 2 and 1 mg/mL were able to reduce biofilm formation (Figure 2(a)). For the preformed biofilm (72 h), the concentrations of 2x MIC (4 mg/mL) and 3x MIC (6 mg/mL) reduced biofilm biomass (Figure 2(b)).

### 3.4. Cell Constituent Release

**Figure 3:**
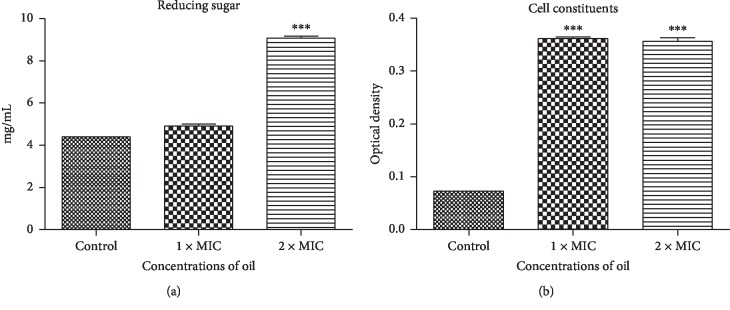
Effect of *Protium heptaphyllum* essential oil (EOPH) on the release of *Vibrio parahaemolyticus* cell constituents. ^*∗∗∗*^*p* < 0.01 vs. all variables.

Figure 3 shows the data for the release of cell constituents: reducing sugar and the absorbance of 260 nm of the supernatants of *V. parahaemolyticus* culture treated with different concentrations of EOPH for 6 h (cell constituents). After treatment with 2x MIC of EOPH, the concentration of reducing sugar increased significantly (*p* < 0.01) from 4.4 to 9.06 mg/mL (2.1 times) and cell constituents (OD_260_) also increased significantly from 0.07 to 0.35 (5 times). On the contrary, after treatment with 1x MIC, only cell constituents (OD_260_) increased significantly (*p* < 0.01) by 5 times.

### 3.5. Cell Membrane Permeability

**Figure 4:**
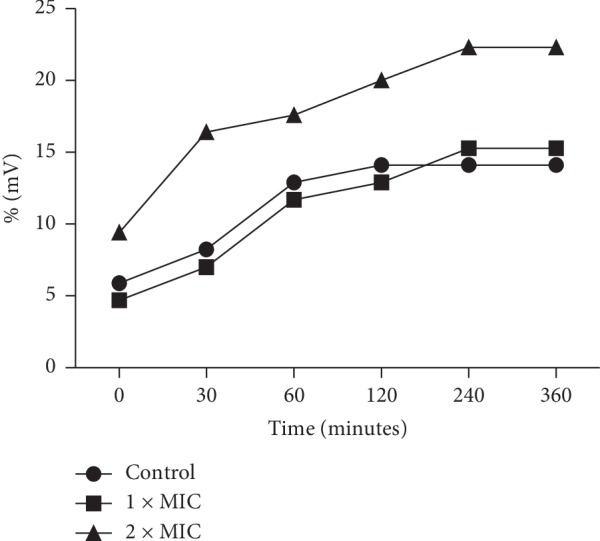
Relative electrical conductivity (% mV) of *Vibrio prahaemolyticus* culture after treatments with *Protium heptaphyllum* essential oil.

The effect of EOPH on the membrane permeability of *V. parahamemolyticus* is presented in Figure 4. The essential oil at 2x MIC concentration, compared to that of the control, influenced the increase of the relative electrical conductivity (% mV) at 30, 60, 120, and 240 minutes, remaining unchanged in the time of 360 minutes. Concentration of 1x MIC only increased the permeability of the membrane at the 240 and 360 minute times when compared with the control, suggesting that in this concentration, the oil only causes lysis and death of bacteria after 4 hours.

## 4. Discussion

*P. heptaphyllum* oleoresins are rich in volatile monoterpenes, exhibiting a chemical composition that can be strongly altered with time [14]. In the present study, the EOPH presented 97.4% of monoterpenes with predominance of *β*-phellandrene (60.68%) and *p*-cymene (13.63%). This chemical characterization differed from previously published reports. EOPH from Ceará (Brazil) presented lower amount of monoterpenes (86.4%), and terpinolene (28.5%) was the main constituent, followed by *α*-pinene (10.5%) and *α*-phellandrene (16.7%) [15]. Mobin et al. [2] reported the main constituents of EOPH determined by gas chromatography-triple quadrupole mass spectrometry were limonene, *p*-cineole, and *o*-cymene. In the EOPH from Acre (Brazil), twenty-one constituents (98.53%) were identified, and *p*-cymene (39.93%) was the main constituent followed by n-tetradecane (13.38%), dihydro-4-carene (11.69%), and *α*-phellandrene (7.41%) [15]. These differences in the chemical composition of essential oils may be due to different factors such as method of extraction and seasonal variations in plant collection site [12].

*β*-phellandrene (3-methylidene-6-propan-2-ylcyclohexene) was the majority component in EOPH (Table 1)—it is a monoterpene of plant origin with several potential commercial applications [16]. In addition, this terpene is naturally synthesized from geranyl diphosphate by a number of plant species and has a commercial value as a key ingredient in medical, cosmetic, and cleaning products [17–19]. The second constituent found in greater quantity in the EOPH was *p*-cymene (1-methyl-4-(1-methylethyl)-benzene) (Table 1) that is a monoterpene with biological activity including antimicrobial effects and found in over 100 plant species. This component is used for medicine and food purposes as a promising candidate to functionalize biomaterials and nanomaterials [19].

The antibacterial activity of EOPH was confirmed by detection of the zone of inhibition (11 ± 0.4 mm) and determination of MIC (2 mg·mL^−1^) and MBC (8 mg·mL^−1^) (Figure 1). The antibacterial effect was confirmed by MIC and MBC values that characterize EOPH as a vibriocidal agent. Lima et al. [3] reported that EOPH showed only activity against Gram-positive bacterium *Streptococcus mutans* (MIC = 0.5 mg·mL^−1^) and did not show antimicrobial activity against *Escherichia coli*, *Staphylococcus aureus*, and *Enterobacter faecalis*. MIC value of the present study was higher than 0.5 mg·mL^−1^, even so it is considered promising because it indicates the action of OEPH against a Gram-negative bacterial species (V. parahemolyticus) with a most complex cell wall chemical composition than gram-positive species and more resistant to the action of essential oils.

The antibacterial activity of other essential oils with *ß*-phellandrene as a major constituent has already been reported indicating the possible relation of this chemical constituent with this biological action. Semeniuc et al. [20] found parsley oil with weak antibacterial activity against *Salmonella enteritidis* (ATCC 13076). However, parsley essential oil with apiol, myristicin, and *β*-phellandrene as major compounds had bactericidal activity against *Staphylococcus aureus* and *Listeria* [21].

The EOPH was effective in reducing the *V. parahaemolyticus* biofilm formation at 4 mg·mL^−1^. In addition, EOPH at 2x MIC (4 mg·mL^−1^) attacked the preformed biofilm after 6 hours of contact with it (Figure 2). These results are promising since *V. parahaemolyticus* is one of the leading food-borne pathogens causing seafood contamination and their biofilm (complex three-dimensional structure supported by extracellular polymeric substances) may constitute a major factor in the dissemination of this bacterium and the ensuing diseases. In addition, frequent monitoring of seafood for *Vibrio* species and their biofilm characteristics is essential to improve seafood safety [22–24].

The results from cell constituent release (Figure 2) and cell membrane permeability (Figure 3) indicate that the concentration of 2x MIC (4 mg·mL^−1^) is capable of causing an irreversible damage to the cytoplasmic membranes and losses of reducing sugar and materials with an absorbance of 260 nm. This confirms the assertion that the constituents of essential oil led to the leakage, disorder, and death by breaking the cell membrane [25].

In addition, it was observed that in the first 30 up to 240 minutes in contact with the bacterial culture, the EOPH at 2x MIC causes an increase in relative electrical conductivity (% mV) indicating damage in the cellular membrane (Figure 3). This is related to the fact that the permeability of the bacterial membrane would have increased correspondingly, causing the leakage of intracellular ingredients, especially losses of electrolytes, including K^+^, Ca^+^, and Na^+^ [12].

## 5. Conclusions

Considering the data of the present research, the EOPH has been confirmed as a potential vibriocidal agent with effect both on inhibition of biofilm formation and on preformed biofilm. Its mechanism of action seems to be related to the increase in cell membrane permeability; however, as the EOPH analyzed in the present study was a mixture of 22 components (Table 1), so it is not possible to specify which of the constituents presented bactericidal activity or even if the mixture of constituents is responsible for the results obtained. Thus, the results of the present research are promising considering that there are still many reports of the EOPH vibriocidal activity.

## Figures and Tables

**Table 1:** Identification and concentration of the *Protium heptaphyllum* essential oil constituents.

Constituents	RI_C_	RI_L_	%
*α*-Pinene	939	940	4.47
*β*-Pinene	979	982	1.54
*cis*-Pinane	986	985	0.73
3-*p*-Menthene	987	1003	0.43
*α*-Phellandrene	1002	1009	3.38
*α*-Terpinene	1017	1022	0.87
*p*-Cymene	1024	1032	13.63
*β*-Phellandrene	1029	1038	60.68
*Γ*-Terpinene	1059	1065	0.13
Terpinolene	1088	1092	0.41
Linalool	1096	1102	0.13
*cis*-*p*-Menth-2-en-1-ol	1121	1127	0.39
Camphor	1146	1143	0.92
Trans-dihydro-*α*-terpineol	1147	1151	1.39
Terpinen-4-ol	1177	1182	0.36
Cryptone	1185	1191	2.59
*α*-Terpineol	1188	1195	3.81
Cuminaldehyde	1241	1246	0.55
Carvone	1243	1250	0.28
Piperitone	1252	1260	0.15
*p*-Menth-1-en-7-al	1275	1280	0.56
*α*-Ylangene	1375	1383	0.47

Total			97.87

RI_C_ = retention index calculated; RI_L_ = retention index in literature.

## Data Availability

The data and materials supporting the conclusions of this article are included within the article.
